# Case Report: Hemoptysis secondary to fish bone migration into the lung parenchyma

**DOI:** 10.3389/fmed.2025.1680237

**Published:** 2025-10-29

**Authors:** Huan Chen, Qi Li, Xinyi Guo, Meiyuan Huang, Nianxi Tan

**Affiliations:** ^1^Central Laboratory, Zhuzhou Central Hospital, Zhuzhou, Hunan, China; ^2^Department of Radiology, Zhuzhou Central Hospital, Zhuzhou, Hunan, China; ^3^Department of Pathology, Zhuzhou Central Hospital, Zhuzhou, Hunan, China; ^4^Department of Cardiothoracic Vascular Surgery, Zhuzhou Central Hospital, Zhuzhou, Hunan, China

**Keywords:** fish bone, hemoptysis, foreign body, thoracoscopic resection, case report

## Abstract

Hemoptysis caused by transmural migration of ingested fish bones into lung parenchyma is exceptionally rare. A 71-years-old diabetic woman presented with 20-days recurrent hemoptysis, exacerbated in supine position. Three months prior, she experienced fish bone impaction with negative laryngoscopy. On re-presentation, contrast-enhanced CT revealed a foreign body embedded in the right upper lobe surrounded by an inflammatory pseudotumor. Thoracoscopic wedge resection confirmed a fish bone intraparenchymal migration with suppurative abscess. The patient recovered uneventfully and remained asymptomatic at 1-month follow-up. This case highlights that even with negative laryngoscopy, patients with fish bone ingestion history require prompt CT to prevent delayed complications. Thoracoscopic resection is effective for pulmonary migration with inflammatory encapsulation.

## Introduction

Hemoptysis typically stems from infections, malignancies, or vascular pathologies. Foreign body aspiration (FBA), though uncommon, demands urgent intervention to avert abscessation or fatal hemorrhage. While most aspirated foreign bodies lodge in the bronchi, transmural migration into lung parenchyma is exceedingly rare, representing a critical diagnostic challenge. Fish bones account for the majority of ingested foreign bodies in Asia. Initial evaluation often involves laryngoscopy, yet its sensitivity for detecting migrated fish bones is low. Computed tomography (CT) has a higher sensitivity than radiography, and enables identification of complications. Delayed diagnosis risks lethal outcomes. We report a case of hemoptysis secondary to fish bone migration into the right upper lobe, emphasizing the imperative for CT despite negative laryngoscopy and the role of thoracoscopic management.

## Case description

A 71-years-old woman visited the outpatient department on October 14th 2024, and complained of recurrent hemoptysis for more than 20 days. She recalled that she had a fish bone lodged in her throat when eating fish at July 27th 2024. On the same day, laryngoscopy examinations conducted at other facilities did not detect any irregularities. She was administered roxithromycin, but no further examinations were made at that point. More than 20 days before presentation, the patient had a paroxysmal hemoptysis. Initially, the patient had hemoptysis in the morning, then it turned into nocturnal hemoptysis, the amount of bleeding ranges from 2 to 4 ml. The symptoms worsened when she lay down and improved when she sat or stood. In 1997, she underwent a radical mastectomy for left breast cancer at the Hunan Cancer Hospital and subsequently received endocrine therapy following the surgery, but did not regularly review. She has a 20-years history of diabetes mellitus and claims to have not used any antidiabetic medication. Her fasting blood sugar levels ranged from 6.1 to 7.0 mmol/L, and postprandial blood sugar levels consistently at 7.0 mmol/L. On December 2nd, 2023, she underwent a vaginal total hysterectomy and an anterior-posterior vaginal wall repair for uterine prolapse. The patient has no history of smoking and no family history of respiratory diseases. She was a retired factory worker living with her daughter. She has limited health literacy, attributing initial hemoptysis to “heatiness.” No occupational dust exposure or recent travel history was reported. At the outpatient clinic, Chest CT revealed a foreign body (FB) surrounded by inflammatory lesions in the upper lobe of the right lung.

**Table:** Timeline table:

Date	Event
1997	Radical mastectomy + endocrine therapy for left breast cancer
2004∼	Diagnosed with DM (no medication); FPG 6.1–7.0 mmol/L, PPG 7.0 mmol/L
2023-12-02	Vaginal hysterectomy + repair for uterine prolapse
2024-07-27	•Fish bone impaction during meal • Laryngoscopy (−) • Roxithromycin prescribed
Late Sep 2024	Paroxysmal hemoptysis; positional worsening
2024-10-14	•Outpatient visit • CT: FB with inflammatory pseudotumor • Hospital admission
2024-10-18	Thoracoscopic wedge resection of lung
2024-10-20	Discharge (post-op day 2)
2024-11-20	1-month follow-up: asymptomatic

### Diagnostic assessment

**FIGURE 1:**
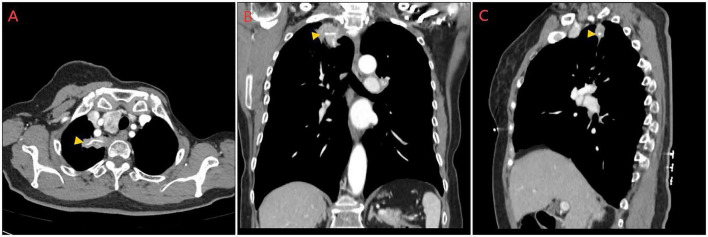
Preoperative contrast-enhanced chest CT images. **(A)** Transverse plane, **(B)** Sagittal plane, and **(C)** Coronal plane views demonstrating a linear, high-attenuation foreign body (fish bone, indicated by orange arrows) embedded within the right upper lobe. The foreign body is surrounded by a focal area of consolidation and ground-glass opacity, consistent with an inflammatory pseudotumor. The multi-planar reconstruction clearly illustrates the intraparenchymal location of the fish bone and its relation to the surrounding lung architecture.

**FIGURE 2:**
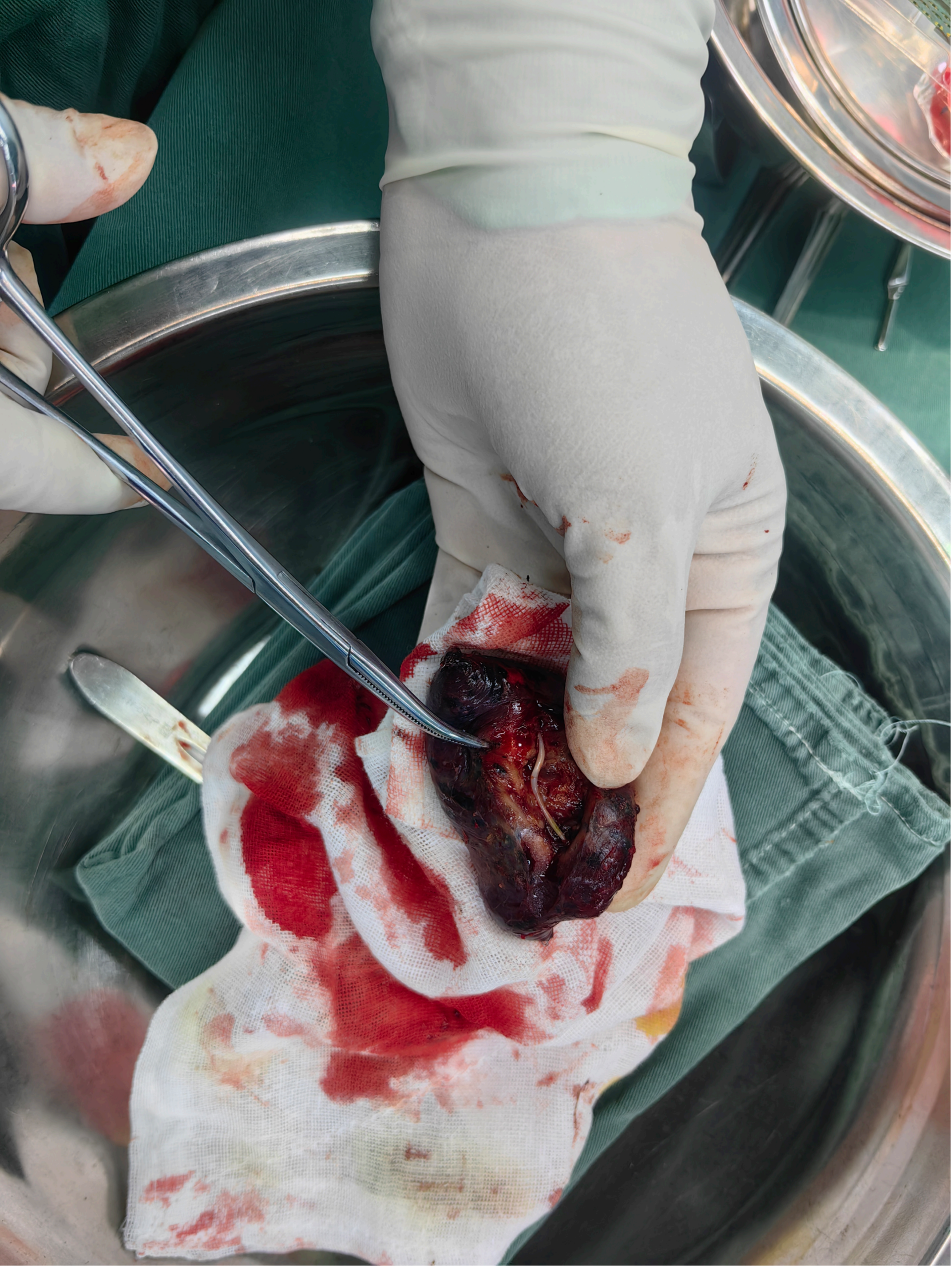
Gross specimen following thoracoscopic wedge resection. The resected segment of the right upper lobe has been bisected, revealing a sharp, slender fish bone measuring approximately 3 cm in length penetrating the lung parenchyma. The surrounding tissue appears firm, hemorrhagic, and discolored, indicative of chronic inflammation and abscess formation.

**FIGURE 3:**
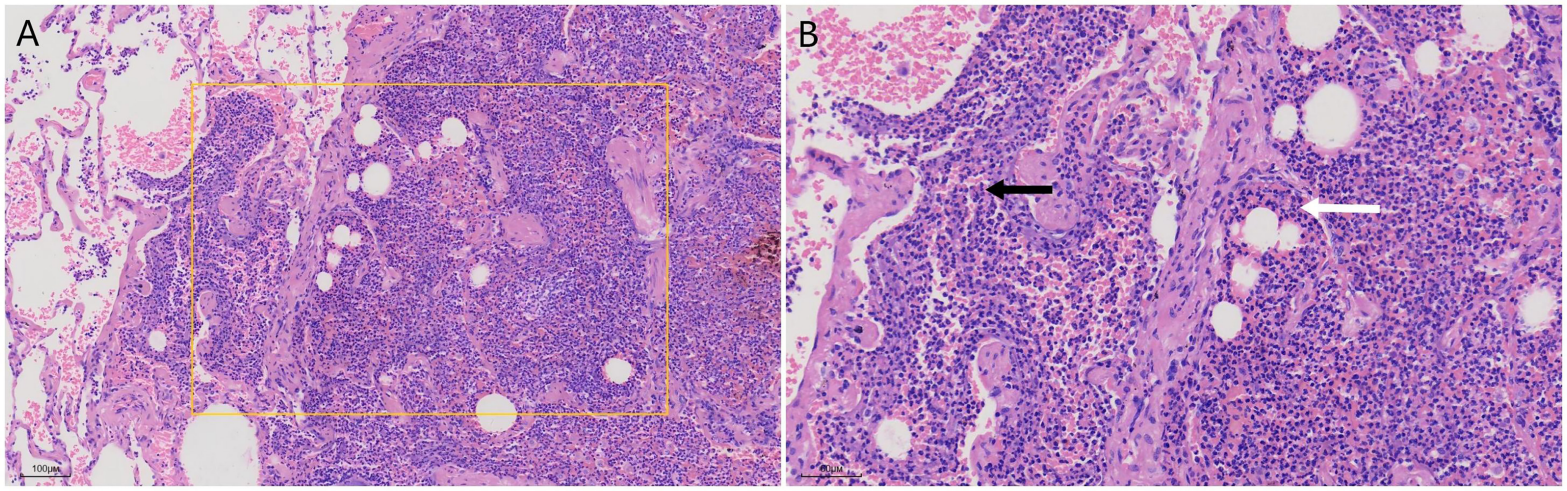
Histopathological examination of the resected lung tissue (Hematoxylin and Eosin staining). **(A)** At low magnification (×40), the architecture of the lung parenchyma is effaced by a dense collection of inflammatory cells forming an abscess (rectangle). **(B)** At higher magnification (×200), the inflammatory infiltrate is predominantly composed of neutrophils (black arrow) and cellular debris, confirming suppurative inflammation. The adjacent alveolar septa are thickened and congested (white arrow).

The patient was directly admitted to the hospital on this same day. Upon hospital admission, pulse, blood pressure, and temperature were normal. The physical examination revealed a barrel chest, diminished respiratory effort, reduced breath sounds, and the presence of wet rales and rhonchi in both lungs. Lab results were within normal parameters except for glycated hemoglobin was 6.7% and uric acid was 383 μmol/L. A repeat contrast-enhanced chest CT still showed the same result (Figure 1), but pneumonia or pulmonary tuberculosis could not be excluded. Tuberculosis of the lungs most commonly occurs in the posterior tip of the upper lobe or the dorsal segment of the lower lobe. Given the history of fish bone impaction, a migrated foreign body was suspected. Upon completion of preoperative investigations, Endoscopic retrieval was not feasible considering that the foreign body is located in the pulmonary parenchyma and surrounded by an inflammatory pseudotumour. On the October 18th 2024, under general anesthesia, a thoracoscopic wedge resection of the right upper lung lobe was performed, with a total operative duration of 110 min. The postoperative isolated specimen confirmed the presence of a 3 cm fish bone embedded in the lung tissue (Figure 2). And the pathological examination revealed lung tissue exhibiting suppurative inflammation and abscess formation (Figure 3). The suppurative inflammation and abscess formation surrounding the fish bone indicated high risk of progressive infection. Without prompt intervention, potential complications included: (1) Abscess expansion leading to pulmonary necrosis or bronchopleural fistula; (2) Hematogenous dissemination causing sepsis, particularly dangerous in this diabetic patient (HbA1c 6.7%); (3) Erosion into pulmonary vessels exacerbating hemoptysis. The embedded location adjacent to the mediastinum further raised concern for mediastinitis, which carries >20% mortality in delayed presentations. Thoracoscopic resection was thus prognostically critical to prevent life-threatening sequelae. The patient experienced a smooth recovery following the surgery and was discharged 2 days later. Tolerability was assessed through: Daily pain scores (NRS consistently ≤ 3/10 with non-opioid analgesia); Documentation of ambulation/spirometer compliance; Normoglycemia maintained via dietary controlling. No treatment-related adverse events occurred. At the 1-month follow-up, she remained asymptomatic. A confirmatory chest radiograph revealed no abnormality. The patient’s delayed presentation (20 days after hemoptysis onset) may including potential factors: (1) No abnormalities were found in laryngoscopy at the first visit and no discomfort symptoms for a long time before the onset of hemoptysis; (2) Cultural perception – attributing symptoms to “heatiness” or age-related decline rather than foreign body complications. These factors underscore challenges in timely diagnosis of occult foreign body migrations in resource-limited populations.

## Discussion

This case illustrates a highly unusual complication of fish bone ingestion: transmural migration into the lung parenchyma causing hemoptysis. While esophageal perforation by fish bones is well-documented, subsequent migration to the lung is exceptionally rare, accounting for only about 3% of thoracic complications (1). Our report underscores the critical importance of maintaining a high index of suspicion in high-risk patients despite initial negative examinations, and highlights the pivotal role of CT imaging and definitive surgical management in such scenarios.

The patient’s profile aligns with established epidemiological data. Fish bones constitute the majority of ingested foreign bodies in Asia (2–6), with older adults, particularly those with dentures or specific eating habits, being at higher risk (4, 7). This 71-years-old diabetic female thus represents a classic high-risk demographic. More critically, she exhibited several independent risk factors for complicated disease, including a long (>2 cm) foreign body, pre-existing diabetes mellitus, and a significant delay between ingestion and presentation with symptoms (2, 8). The confluence of these factors likely contributed to the severe sequela of transmural migration.

**FIGURE 4:**
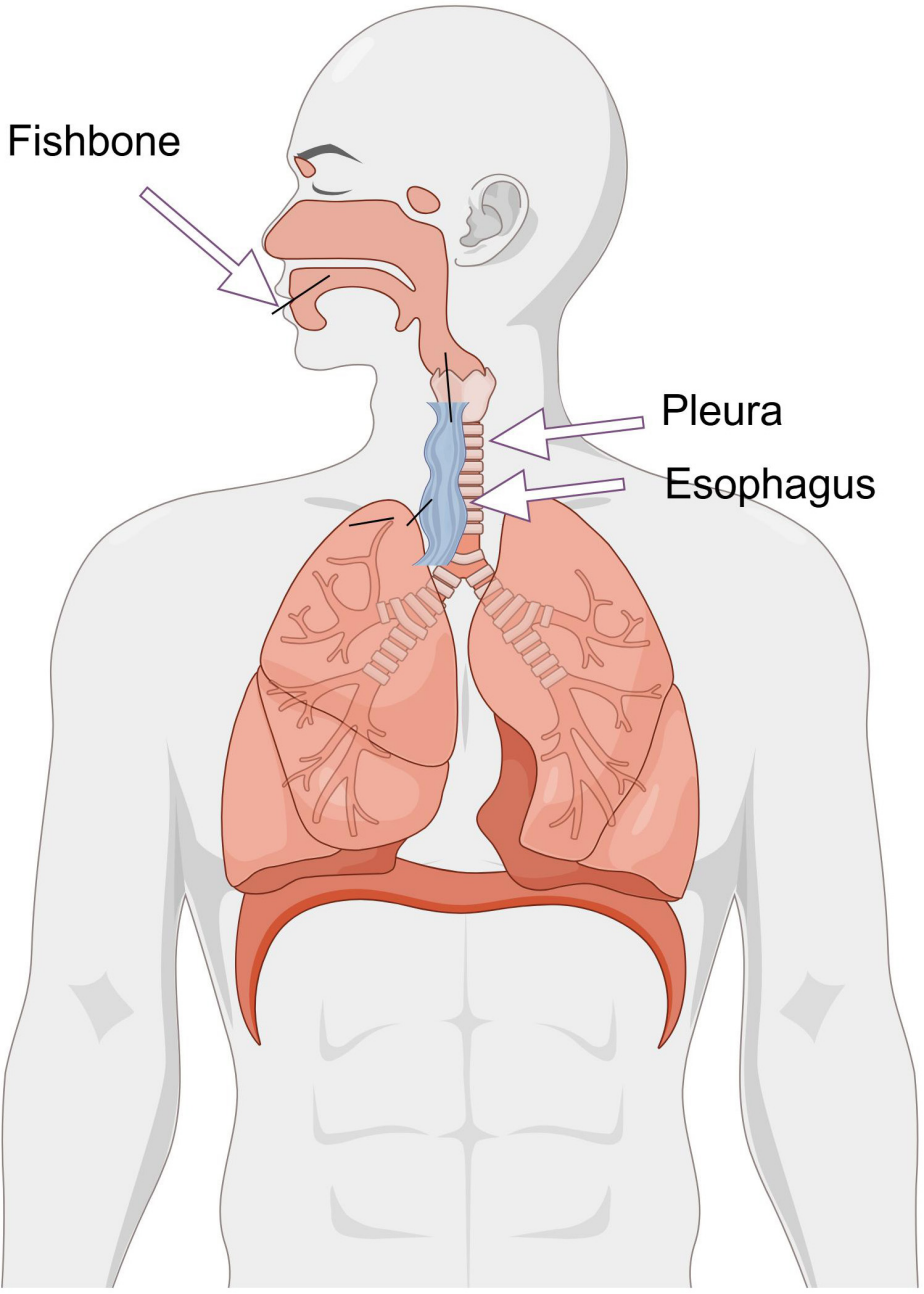
Proposed mechanism of fish bone migration from the esophagus to the lung parenchyma. The diagram illustrates the hypothesized pathway: (1) The fish bone enters the esophagus through the mouth. (2) Impaction and perforation of the esophageal wall by the ingested fish bone, transmediastinal migration through the loose connective tissue. (3) Final penetration into the parenchyma of the right upper lobe, leading to abscess formation and hemoptysis.

The fish bone ectopically located in the patient’s lung parenchyma is the postcleithrum. This type of bone is pointed at both ends, spoon-shaped, and S-curved. It is more likely to migrate to the organs around the esophagus under the influence of external forces. The mechanism was that the fish bone penetrated the esophageal wall and migrated to the upper lobe of the right lung through the mediastinum (Figure 4).

The initial negative laryngoscopy following ingestion provided false reassurance, leading to a diagnostic delay of over 2 months. This case powerfully demonstrates the limitations of laryngoscopy and plain radiographs in detecting migrated foreign bodies (9–11). As evidenced here, computed tomography (CT) is the undisputed gold standard for diagnosis. It not only identified the ectopic fish bone with high sensitivity (10, 12) but also characterized its intraparenchymal location and the surrounding inflammatory response (inflammatory pseudotumor), which was crucial for preoperative planning. This advantage holds even with low-dose protocols (13), reinforcing CT’s role as the first-line imaging modality when there is a clear history of ingestion, irrespective of initial laryngoscopic findings (14, 15).

Beyond technical factors, the diagnostic delay may also be attributed to the patient’s health beliefs. Her attribution of hemoptysis to “heatiness” reflects a cultural perception that can divert attention from serious organic pathology. This highlights a broader challenge in clinical practice: the need for clinicians to actively explore patients’ explanatory models for their symptoms and provide clear education on “red-flag” signs that necessitate rigorous biomedical investigation.

Endoscopic retrieval is the cornerstone of management for intraluminal foreign bodies (3, 5, 16). However, when the object migrates extraluminally into the pulmonary parenchyma, as confirmed in our case, endoscopic intervention becomes futile and surgery is mandated (17). The choice of thoracoscopic wedge resection was optimal. It is a minimally invasive approach that allows for direct visualization, complete removal of the foreign body, and resection of the surrounding septic focus (the suppurative abscess), thereby preventing catastrophic sequelae such as massive hemoptysis, bronchopleural fistula, or mediastinitis (18). Our patient’s rapid recovery underscores the efficacy and tolerability of this approach, even in patients with comorbidities.

In conclusion, we present a rare case of hemoptysis secondary to pulmonary migration of a fish bone. This report serves as a critical reminder that: A negative initial laryngoscopy does not rule out a migrated foreign body; A detailed history of ingestion must trigger timely and advanced imaging, with CT being the investigation of choice; Surgical intervention, particularly thoracoscopic resection, is a safe and effective definitive treatment for Lung Parenchyma foreign bodies with associated infection. Clinicians should be aware of the potential for atypical presentations and migrations, especially in high-risk patients, to prevent life-threatening delays in diagnosis and treatment. As a single-center case report, our conclusions are constrained by the inherent limitations of retrospective documentation. The extreme rarity of pulmonary migration limits generalizability, and long-term outcomes beyond 1 month require further monitoring. Nevertheless, this case provides valuable insights into the management of a complex and easily missed clinical entity.

## Patient perspective

In her own words during the 1-month follow-up, the patient described her experience:

“The constant fear of choking on blood while lying down made me dread nights the most. I felt relieved when the CT scan finally found the cause after weeks of uncertainty. Before surgery, I worried about big scars and slow recovery because of my past breast cancer operation, but the doctors explained that this would be a “keyhole surgery.” Waking up with only three small bandages surprised me – the pain was manageable with oral medications. By the next morning, I could walk with support. What mattered most was that the bloody cough disappeared immediately after surgery. I now understand that even small symptoms need timely checks, especially for diabetics like me.”

She emphasized the value of clear communication from the medical team regarding the migration mechanism and postoperative diabetic management.

## Data Availability

The original contributions presented in this study are included in this article/supplementary material, further inquiries can be directed to the corresponding author.
